# Morphological features of bronchiectasis in patients with non-tuberculous mycobacteriosis and interstitial pneumonia

**DOI:** 10.1186/s13104-022-06156-3

**Published:** 2022-07-26

**Authors:** Chiori Tabe, Masaki Dobashi, Yoshiko Ishioka, Masamichi Itoga, Hisashi Tanaka, Kageaki Taima, Sadatomo Tasaka

**Affiliations:** Department of Respiratory Medicine, Hirosaki University Graduate School of Medicine, Hirosaki, 036-8562 Japan

**Keywords:** 3D image, Bronchial structure, Bronchiectasis, Idiopathic pulmonary fibrosis, Non-tuberculous mycobacteriosis

## Abstract

**Objective:**

To compare the morphological features of bronchiectasis between patients with different underlying diseases, we performed quantitative analysis of high-resolution computed tomography (HRCT) images of 14 patients with non-tuberculous mycobacteriosis (NTM) and 13 with idiopathic pulmonary fibrosis (IPF). A 3D image of the bronchial structure was made from HRCT data. Bronchiectasis was defined as abnormal dilatation of the bronchi with the diameter greater than that of the accompanying pulmonary artery. We measured the inner and outer diameters, wall area as %total airway cross sectional area (WA%), and wall thickness to airway diameter ratio (T/D) of the 4-8th generations of bronchi.

**Results:**

In patients with IPF, the inner and outer diameters linearly decreased toward the distal bronchi. In contrast, the inner and outer diameters of NTM fluctuated. The coefficient of variation of the outer diameters of the 6-7th generations of bronchi was larger in the NTM patients than in those with IPF, whereas no significant difference was observed in the coefficient of variation of the inner diameters between the groups. In IPF patients, WA% and T/D varied between the generation of bronchi, but the coefficient of variation of WA% and T/D was relatively small in those with NTM.

## Introduction

Bronchiectasis is a progressive respiratory disease characterized by irreversible and pathological dilatation of the small- and medium-sized bronchi [1]. In recent years, the prevalence of bronchiectasis has increased due to the aging of the population and the widespread use of high-resolution computed tomography (HRCT), and the high morbidity and mortality rates have made it a worldwide problem [2].

The etiology of bronchiectasis can be divided into two patterns. One is mainly caused by chronic or recurrent infections or other chronic airway inflammation, which causes loss of smooth muscle and elastic, leading to airway remodeling [3, 4]. *Mycobacterium avium* complex (MAC) and other non-tuberculous mycobacteria are known to be the major etiologic organisms of bronchiectasis and account for a large proportion of cases in Japan [5]. The other is traction bronchiectasis, which is caused by collapse of the surrounding alveoli due to inflammation or fibrosis. Traction bronchiectasis refers to the irreversible dilatation of the bronchi and is known as an important feature of fibrotic lung disease [6]. Thus, bronchiectasis has different origins and pathological findings depending on the etiology, but little is known about the morphological features of bronchiectasis of different etiologies.

In 1980’s, computed tomography (CT) is applied to diagnosis of bronchiectasis with sensitivities of 60–100% and specificities of 92–100% [7]. Recent advances in imaging technology, such as thickness and interval of sclices, improved the diagnostic accuracy, and HRCT is the standard tool of the diagnosis of bronchiectasis [1]. However, no report has quantitatively evaluated the morphological differences between bronchiectasis due to airway remodeling and traction bronchiectasis using imaging data.

In the present study, we aimed to quantitatively compare the morphological characteristics between bronchiectasis due to nontuberculous mycobacteriosis (NTM) and traction bronchiectasis associated with idiopathic pulmonary fibrosis (IPF) by using CT workstation and 3D-images of HRCT.

## Main text

### Methods

#### Study subjects

This is a retrospective observational study and its protocol was approved by the Ethics Committee of Hirosaki University Graduate School of Medicine (approval number: 2019–1043). Written informed consent was waived because of the retrospective design of the study. We evaluated consecutive patients who were treated for NTM or IPF between January 2014 and December 2018. The diagnosis of NTM lung disease was made by the ATS/IDSA diagnostic criteria published in 2007 [8]. For the diagnosis of IPF, the 2018 updated guideline was used [9]. Patients who did not undergo HRCT with a slice thickness of 1.0 mm or less and those who had no findings of bronchiectasis were excluded.

#### Morphological analysis

We evaluated the HRCT images using a CT-3D imaging workstation (Ziostation2^®^, Ziosoft, Tokyo, Japan). On HRCT, bronchiectasis was defined as abnormal dilatation of the bronchi with the diameter greater than that of the accompanying pulmonary artery [10]. We measured the inner and outer diameters, wall area as %total airway cross sectional area (WA%), and wall thickness to airway diameter ratio (T/D) of the 4th-8th generations of bronchi [11].

**Fig. 1:**
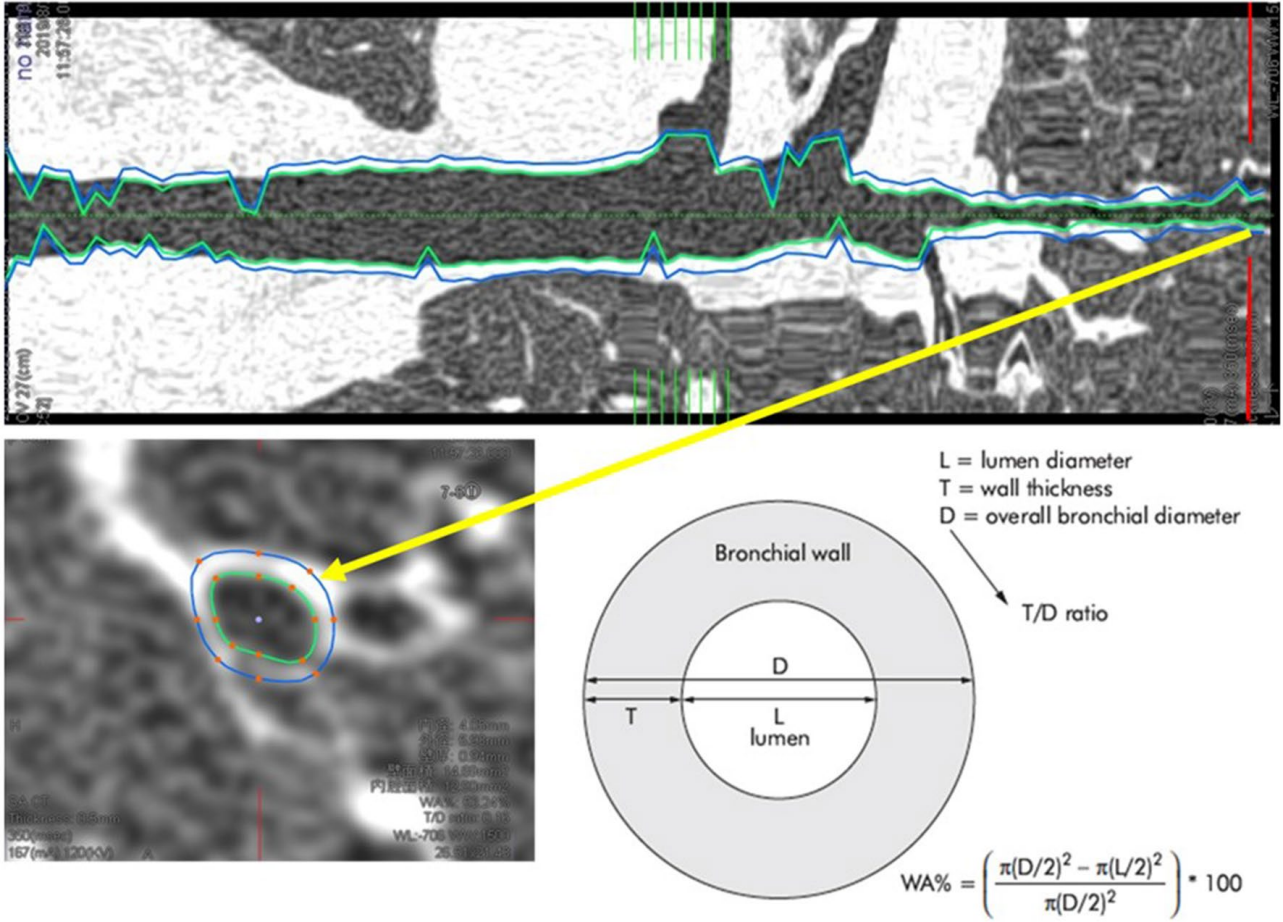
Measurement procedure using a CT-3D imaging workstation. The bronchus to be measured was selected on the left CT image, and a bronchial pathway was created by automatic extraction (*top*). The bronchial cross section at the specified position is displayed and the inner and outer diameters are marked (*bottom left*). The inner and outer diameters, WA%, and T/D ratio were automatically calculated for the 4th-8th generations of bronchi (*bottom right*). L: lumen diameter, T: wall thickness, D: overall bronchial diameter. In the measurement screen, multiple measurements of T, D, and L are made, and their average are calculated

As for the measurement procedure, we first constructed 3D images from the captured HRCT images and selected a bronchus to be measured. The path from the trachea to the target bronchus was automatically extracted. In cases the lung structure was highly deformed and automatic extraction by the application was difficult, manual plots from the trachea to bronchus were performed (Fig. 1). Once the bronchi are extracted, the bronchial cross section at the specified position is displayed and the inner and outer diameters are marked. Once the marking was confirmed, the inner and outer diameters, WA%, and T/D ratio were automatically calculated for the 4th-8th generations of bronchi. Since intrapulmonary structures were distinguished by CT values, manual corrections were made when there was a large discrepancy between the outer and inner wall markings. Three points were measured for each generation of bronchial tubes, and the average of these points was used to calculate the value. When the distance to the branch of the bronchus was too short to obtain three measurement points, one or two points were measured and the average was calculated.

#### Statistics

Data were presented as means and standard deviations, and the coefficient of variation was calculated by dividing the standard deviation by the mean value. Statistical analyses were performed using JMP 15 (SAS Institute, Cary, NC, USA).

## Results

### Patient characteristics

A total 27 patients, 14 with NTM and 13 with IPF, were included in this study. All of the IPF patients were diagnosed solely with HRCT that revealed a usual interstitial pneumonia (UIP) pattern in 12 patients and a probable UIP pattern in one. None of the study subjects underwent lung biopsy.

The mean age of the patients with NTM was 76.2 ± 6.7 years; 27% were male, 14% had smoking history. On the other hand, the mean age of the IPF patients was 68.9 ± 8.1 years; 77% were male, 92% had smoking history. There was no significant difference in age between the patients with NTM and those with IPF. In terms of gender, there were more females in the NTM group and more males in the IPF group. Most of the IPF patients were smokers. The lung function data showed decreases in %FVC (58.1 ± 14.6%), and %DL_CO_ (46.9 ± 18.1%), suggesting that most of the IPF patients included had moderate or severe disease.

### Morphological parameters

**Fig. 2:**
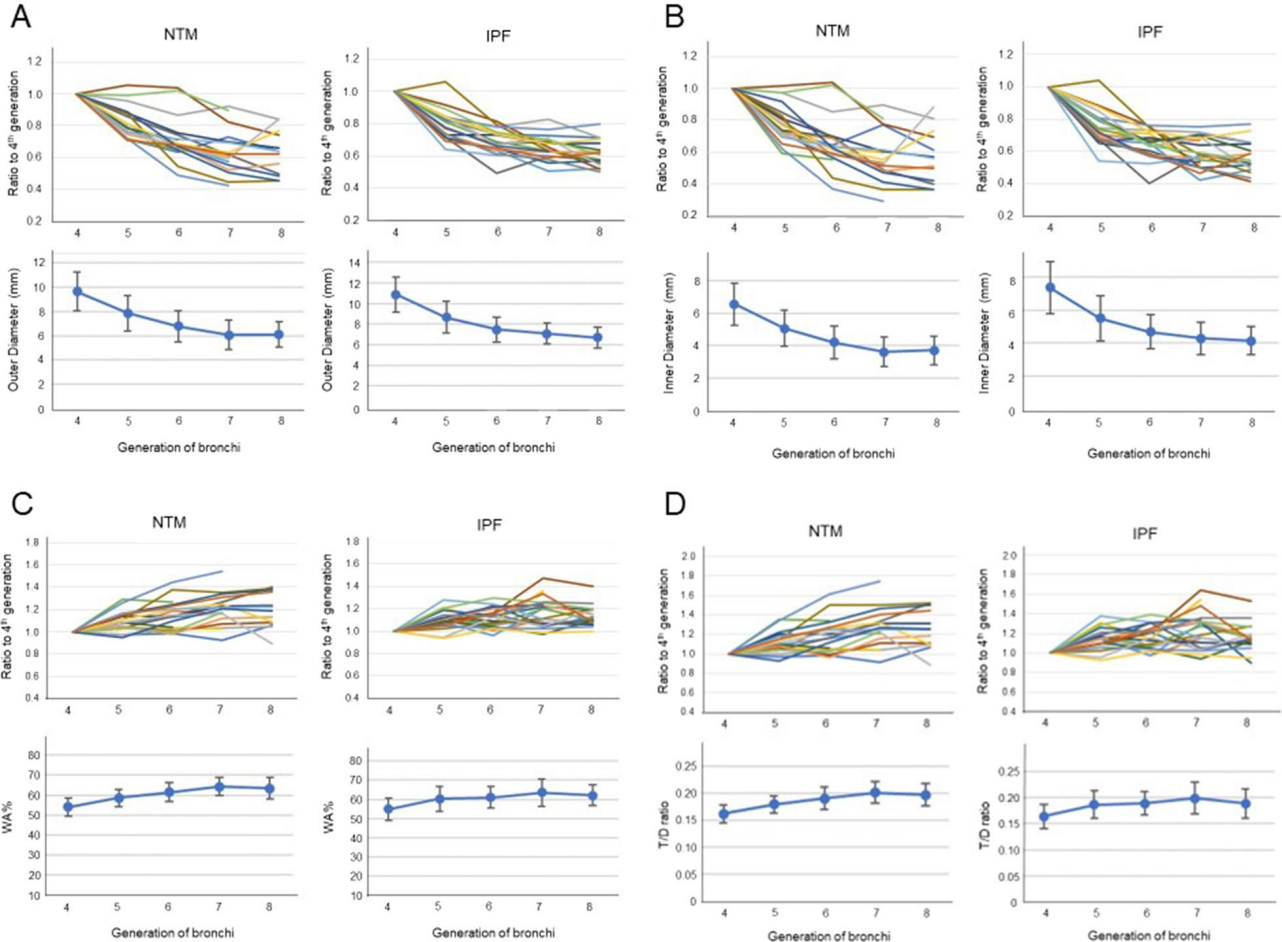
Morphological parameters of bronchiectasis. **A** Outer diameter, **B** Inner diameter. In the patients with IPF, the outer and inner diameters decreased linearly toward the distal part of the bronchus. In contrast, some bronchi were larger at the distal part, and the outer and inner diameter measurements of a generation of the bronchi were largely different between the patients with NTM. **C** WA%, **D** T/D ratio. WA% and T/D ratio in most of the NTM patients increased linearly toward the distal part of the bronchus although the values were diverse between the subjects. In the patients with IPF, larger values of WA% and T/D ratio were observed at the distal part

The morphological parameters of bronchiectasis evaluated in this study were summarized in Fig. 2. Line graphs were made for four items: inner diameter, outer diameter, WA%, and T/D ratio. The graphs were created by calculating the ratios of the measurements for the 5th to 8th generations of bronchi, using the 4th generation bronchi as a reference.

In the patients with IPF, the outer and inner diameters decreased linearly toward the distal part of the bronchus (Fig. 2A, B). In contrast, in the NTM patients, some bronchi were larger at the distal part, and the outer and inner diameter measurements of a generation of the bronchi were largely different between the patients.

Contrary to the results for outer and inner diameter, WA% and T/D ratio in most of the NTM patients increased linearly toward the distal part of the bronchus although the values were diverse between the subjects (Fig. 2C, D). In the patients with IPF, larger values of WA% and T/D ratio were observed at the distal part.

#### Coefficient of variation of the morphological parameters

Since the results of the line graph showed that there was a large variation in the measurements between the 5th and 7th generations of the bronchi, the coefficient of variation was calculated (Table 1). In the 6th to 7th generations of the bronchi, the coefficient of variation of the outer diameter was larger in the NTM patients than in those with IPF. The coefficients of variation of the inner diameter were similar in the NTM and IPF patients. The coefficients of variations of WA% and T/D were relatively small in the NTM patients, compared to those in the IPF patients.

**Table 1:** Coefficient of variation

	Generation of bronchi	NTM (n = 14)	IPF (n = 13)
Outer Diameters	6th	0.1908	0.1656
	7th	0.2005	0.1441
Inner Diameters	6th	0.2377	0.2213
	7th	0.2443	0.2256
WA%	6th	0.0799	0.0909
	7th	0.0700	0.1145
T/D ratio	6th	0.1067	0.1172
	7th	0.0962	0.1508

*WA%* wall area as % total airway cross sectional area

*T/D ratio* wall thickness to airway diameter ratio

### Discussion

**Fig. 3:**
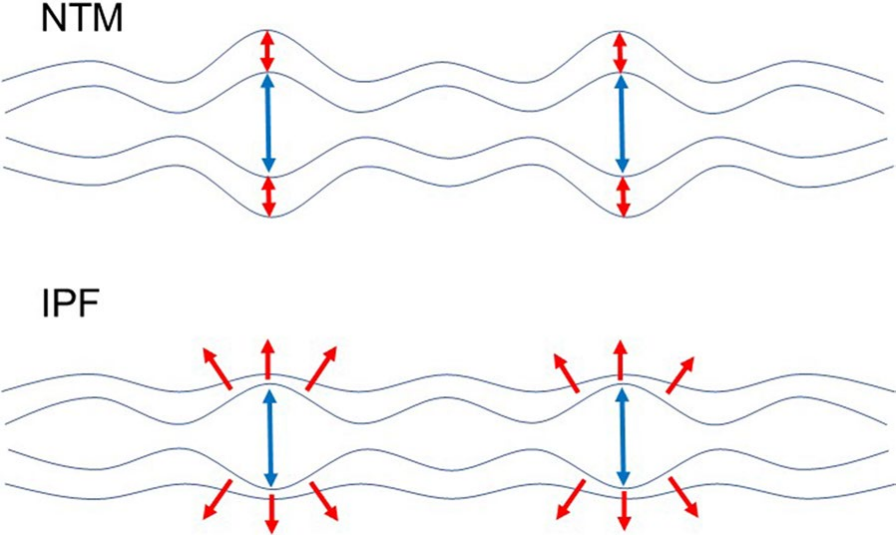
Schematic diagram of morphological features of bronchiectasis. Bronchiectasis in NTM patients has a larger lumen and thicker walls due to edematous changes of the bronchial wall, whereas IPF patients have a larger lumen and thinner airway wall due to traction along with the fibrosis of the surrounding alveoli

In the presents study, morphological features of bronchiectasis were evaluated using HRCT images, comparing the patients with NTM and those with IPF. In the NTM patients, the inner diameter and outer diameter fluctuated greatly depending on the generation of the bronchus, while in the IPF patients, the inner diameter and outer diameter decreased linearly toward the distal side. In contrast, in the NTM patients, WA% and T/D ratio increased linearly toward the distal side, whereas, in the IPF patients, WA% and T/D ratio fluctuated between generations of the bronchi. In summary, the changes seen in dilated bronchi differed between NTM and IPF, with NTM having a larger lumen and thicker wall possibly due to edematous changes of the bronchial wall, and IPF having a larger lumen and thinner airway wall due to traction along with the fibrosis of the surrounding alveoli. (Fig. 3). To the best of our knowledge, this is the first report that evaluated morphological features of bronchiectasis using HRCT images.

Lung disease, which accounts for 80–90% of NTM, is a chronic progressive disease, causing bronchiectasis [12]. Its characteristic pathologic findings include extensive granulomas affecting the airways. Peribronchial granulomas of the trachea, bronchi, and bronchioles can cause airway narrowing, and at the same time, granulomas can disrupt the muscular layer of the airway and cause bronchiectasis [13, 14]. The increased thickness of the tracheal wall seen in NTM may also reflect ulceration of the bronchial wall and edematous changes due to infiltration of inflammatory cells into the periphery of the bronchi.

On the other hand, traction bronchiectasis is a characteristic finding in fibrotic lung diseases such as IPF. Pathologically, it is a secondary dilatation of the bronchi and bronchioles as a result of contraction of the lung tissue around the airways due to inflammation, fibrosis and scarring [7]. The presence of traction bronchiectasis in IPF has been reported to correlate with the abundance of fibroblastic foci [15], which is also a predictor of poor prognosis [16]. In addition, in the UIP pattern seen in IPF, fibrosis has been found to begin in the alveolar structures and continue from the peripheral lobular margins into the more proximal airways [17].

In this study, we observed that the IPF patients had dilated bronchi with stretched and thinned walls, which were consistent with the pathological findings. However, there was no significant difference in the coefficient of variation between the distal and proximal sides, probably because we measured bronchiectasis that had already been completed after a long period of time. If the measurements had been taken at a relatively early stage of the disease, there might have been a difference between distal and proximal bronchiectatic findings.

In the present study, we included patients with NTM as a representative of chronic lower respiratory tract infections. However, NTM is caused by intracellular organisms and pathologically characterized by granulomas, which is different from most bacterial infection [18]. It remains unclear whether the results of this study can be applicable to chronic lower respiratory tract infections caused by other pathogens, such as *Haemophilus influenzae* and *Pseudomonas aeruginosa*.

There have been a few reports about the distribution of bronchiectasis, and the spatial heterogeneity of the disease makes it difficult to reach a consensus. Reid described that, in cylindrical bronchiectasis, the average numbers of bronchial subdivisions were 7.5 on bronchogram but 16 in histopathology [19]. Recently, Ikezoe and colleagues reported that thickening of the airway wall and dilation of the lumen were observed in the 7th to 17th generation in IPF patients [20]. They also noted that the airway lumen was dilated due to the presence of markedly non-uniform strain along the bronchioles. This may be comparable with the coefficient of variation used as a measure of variability in our study.

## Conclusions

The present study indicated that the morphological features of bronchiectasis differ between NTM and IPF patients: edematous changes in the bronchial wall in NTM and tractional thinning of the bronchial wall in IPF, each condition may be reflected in the imaging findings. For clinical application, our method will be helpful when considering a diagnostic approach for patients with bronchiectasis such as intensive microbiological tests or histological examination with interstitial pneumonia in mind.

## Limitations

There are several limitations to our study. Firstly, the study was conducted at only one institution and the total number of patients was small. The existence and effect of chance errors cannot be denied. Secondly, the automatic extraction of the airway by the image workstations is based on the CT values, which makes it difficult to distinguish between anatomical airway structures and transient appearance of secretions or other materials when their CT values are similar. Thirdly, we evaluated the HRCT data taken at a single time point in the patients with established bronchiectasis. Due to the lack of longitudinal data, it remains unclear whether the results of the present study can be applicable to the airways with developing bronchiectasis.

## Data Availability

The datasets used and analyzed during the current study are available from the corresponding author on reasonable request.

## Abbreviations

CT: Computed tomography
HRCT: High-resolution computed tomography
IPF: Idiopathic pulmonary fibrosis
MAC: *Mycobacterium avium* Complex
NTM: Non-tuberculous mycobacteriosis
T/D: Wall thickness to airway diameter ratio
WA%: %Total airway cross sectional area
